# Synapses as Therapeutic Targets for Autism Spectrum Disorders: An International Symposium Held in Pavia on July 4th, 2014

**DOI:** 10.3389/fncel.2014.00309

**Published:** 2014-09-29

**Authors:** Paolo Curatolo, Yehezkel Ben-Ari, Yuri Bozzi, Maria Vincenza Catania, Egidio D’Angelo, Lisa Mapelli, Lindsay M. Oberman, Christian Rosenmund, Enrico Cherubini

**Affiliations:** ^1^Pediatric Neurology Unit, Department of Neurosciences, Tor Vergata University, Rome, Italy; ^2^Institut National de la Santé et de la Recherche Médicale, Mediterranean Institute of Neurobiology (INMED), Marseille, France; ^3^CNR Neuroscience Institute and Laboratory of Molecular Neuropathology, Centre for Integrative Biology (CIBIO), University of Trento, Trento, Italy; ^4^CNR, Institute of Neurological Sciences (ISN), Catania, Italy; ^5^Laboratory of Neurobiology, Istituto di Ricovero e Cura a Carattere Scientifico Oasi Maria SS, Troina, Italy; ^6^Department of Brain and Behavioral Sciences, University of Pavia, Pavia, Italy; ^7^Brain Connectivity Center, Neurological Institute Istituto di Ricovero e Cura a Carattere Scientifico Mondino, Pavia, Italy; ^8^Department of Psychiatry and Human Behavior, Warren Alpert Medical School, Brown University, Providence, RI, USA; ^9^Neuroscience Research Center and NeuroCure Cluster of Excellence, Charité-Universitätsmedizin Berlin, Berlin, Germany; ^10^International School for Advanced Studies (SISSA), Trieste, Italy; ^11^European Brain Research Institute (EBRI), Rome, Italy

**Keywords:** ASDs, animal models, synapse, therapeutic target, GABAergic signaling

## Abstract

New progresses into the molecular and cellular mechanisms of autism spectrum disorders (ASDs) have been discussed in 1 day international symposium held in Pavia (Italy) on July 4th, 2014 entitled “synapses as therapeutic targets for autism spectrum disorders” (satellite of the FENS Forum for Neuroscience, Milan, 2014). In particular, world experts in the field have highlighted how animal models of ASDs have greatly advanced our understanding of the molecular pathways involved in synaptic dysfunction leading sometimes to “synaptic clinical trials” in children.

Autism comprises a wide range of neuro-developmental disorders known as autism spectrum disorders (ASDs), characterized by deficits in verbal and non-verbal communication, impaired social interactions, restricted interest, and stereotyped behavior. ASDs affect 1 over 150/200 children, with onset before 3 years of age. The higher incidence of these disorders in recent years can be attributed to the improvement of the diagnostic criteria in general and to the increased attention of the medical community (Geschwind and Levitt, 2007). Unlike idiopathic forms, syndromic forms constitute only 10% of cases often associated with malformations and/or dysmorphic features. Syndromic forms originate from well-known genetic or genomic disorders (Lintas and Persico, 2009). These include fragile X, Angelman, Rett syndromes, neurofibromatosis, tuberous sclerosis, etc. (Benvenuto et al., 2009). Interestingly, evidence has been provided that, in a small number of cases, single mutations in genes encoding for synaptic proteins account for behavioral deficits observed in autistic children. Although rare, these new discoveries point to synapses as possible sites of autism’s origin (Südhof, 2008). In particular, the neurotransmitter GABA has emerged as one of the major players in the assembly and formation of neuronal circuits early in development (Ben-Ari et al., 2012), and deficits in GABAergic signaling are instrumental in the genesis of ASD as demonstrated by the high co-morbidity of these disorders with epilepsy.

In order to identify the molecular and cellular mechanisms underlying ASDs, several animal models have been generated over the last decade. To this aim, genetic defects detected in patients have been introduced in the mouse genome. Animal studies have revealed important dysfunctions in synaptic signaling associated with behavioral deficits reminiscent of those found in autistic children. This has allowed to develop new tools for the treatment of ASDs opening new perspectives for early therapeutic interventions.

To highlight recent progress in these specific points, leading experts in the field gathered together in Pavia on July 4th 2014 for 1 day symposium (satellite of the FENS Forum) entitled “synapses as therapeutic targets for autism spectrum disorders.” We discussed how animal models greatly advanced our understanding of the molecular pathways involved in synaptic dysfunction in ASDs leading sometimes to “synaptic clinical trials” in children.

Christian Rosenmund (Berlin, Germany) talked about current work obtained in his laboratory on the cellular mechanisms underlying the Rett syndrome (RTT), a postnatal neurological disorder affecting 1/10000 girls. RTT is caused by loss of function mutations in the X-linked gene encoding the transcriptional repressor, methyl-CpG binding protein 2 (MECP2). Rett patients exhibit impaired motor coordination, learning deficits, irregular breathing, and seizures. Recent findings show that over-expression of wild-type (WT) MeCP2, due to gene duplications, causes also mental retardation and progressive neurological symptoms in males. Neurological phenotypes involve an imbalance of excitation and inhibition in neural circuits caused by too little or too much MeCP2 activity. Using a reduced experimental system consisting in growing single neurons on micro-island cultures, he provided evidence for specific determination of cell intrinsic mechanisms of MeCP2 regulation (Chao et al., 2007, 2010). The cellular expression levels of MeCP2 directly regulate synapse formation in cultured cortical excitatory neurons, and the underlying mechanism seems to be related to impaired autocrine BDNF function. These results form the basis of future studies to develop putative rectifying treatments to regain balance of excitation and inhibition in RETT patients.

Maria Vincenza Catania (Catania, Italy) briefly synthesized the state of the art research supporting the notion that dysfunctions of group-I metabotropic glutamate receptor 5 (mGlu5) are implicated in the pathophysiology of fragile X syndrome (FXS), the most common form of inherited intellectual disability and an important monogenic cause of autism. In most cases, FXS is caused by mutations in the fragile X mental retardation gene 1 (FMR1), silencing of the gene and ensuing lack of the encoded fragile X mental retardation protein (FMRP), an RNA binding protein involved in RNA metabolism and protein synthesis. FMRP is present at synapses and controls local mRNA translation upon synaptic activity. Although much work has focused on dysregulation of mGlu5 signaling to synaptic protein synthesis, emerging evidence finds abnormal mGlu5 receptor interactions with its scaffolding Homer protein, which results in mGlu5 receptor dysfunction and phenotypes independent of signaling to protein synthesis (D’Antoni et al., 2014). In particular, in the brain of Fmr1 knock-out mice, mGlu5 receptors are less associated to the constitutive forms of Homer proteins (Giuffrida et al., 2005; Ronesi et al., 2012). In the absence of FMRP, several properties and functions of mGlu5 receptors are altered, partly as a consequence of mGlu5/Homer disruption. She discussed the implication of these findings for the pathophysiology and therapeutic correction of FXS.

Paolo Curatolo (Rome, Italy) described recent data on tuberous sclerosis complex (TSC), caused by haploinsufficiency of the *TSC1* and *TSC2* genes. This leads to over-activation of the mTORC1-eIF4E pathway, known to regulate growth and synaptic plasticity of glutamatergic neurons with alterations of synaptic homeostasis. Although the exact mechanisms of ASDs in TSC are still unclear, changes in the expression of specific AMPA and GABA_A_ receptor subunits, as well as loss of GABAergic interneurons have been described (Curatolo et al., 2010). Interestingly, mouse models of TSC have demonstrated that the behavioral phenotype may be the direct consequence of the *TSC1/TSC2* dysfunction. Pharmacological inhibition of the mTOR pathway with rapamycin is able to restore the excitatory/inhibitory balance and to reverse the autistic-like behavior of *TSC2*^(+/−)^ mice (Sato et al., 2012). Furthermore, both heterozygous and homozygous loss of *TSC1* in mouse cerebellar Purkinje cells (PC) results in autistic-like behavior associated with a decreased in PC excitability. These effects can be prevented by rapamycin, suggesting a possible therapeutic role for mTOR inhibition in TSC-related ASDs (Tsai et al., 2012). As a whole, these findings suggest that mTOR over-activation contributes to behavioral phenotypes of TSC-related autism and mTOR inhibitors offer potential therapeutic avenues for the pharmacological treatment of ASD-associated with mTORpathies.

Lisa Mapelli (from Egidio D’Angelo’s group, Pavia, Italy) discussed modifications of cerebellar circuits in an animal model of the Phelan–McDermid syndrome. This autistic syndrome is caused by a deletion of the terminal portion of chromosome 22 (22q13.3) that involves both *Shank3* and *Ib2* genes. The latter encodes for the synaptic protein IB2 that takes part to the NMDA receptor interactome. Electrophysiological recordings combined with voltage-sensitive dye imaging in *Ib2* KO mice (Giza et al., 2010) unveiled a three to fivefold increase in NMDA receptor-mediated currents in cerebellar granule cells associated with an altered spatial distribution of excitation and inhibition in the granular layer (Mapelli et al. unpublished data). The altered excitatory/inhibitory balance was accompanied by changes in amplitude and spatial organization of long-term potentiation (LTP) and long-term depression (LTD) at mossy fiber-granule cell synapses. These changes, reminiscent of those reported in other animal models of ASD (Casanova, 2006) suggest common alterations in neuronal network activity in different brain areas.

Enrico Cherubini (Trieste, Italy) illustrated the neuroligin (NL) model generated in Südhof’s laboratory by introducing in mice a single mutation (R451C) of the human *Nlgn3* gene detected in a family with children affected by ASDs (Tabuchi et al., 2007). NLs are post-synaptic adhesion molecules that bind to their presynaptic partners neurexins to functionally couple the post-synaptic densities with the transmitter release machinery. Mice carrying the NL3^R451C^ mutation show modifications of GABAergic signaling associated with social deficits reminiscent of those found in autistic children. A detailed analysis of GABAergic microcircuits in the hippocampus has unveiled an increased GABA release from cholecystokinin-positive endocannabinoid-sensitive interneurons and a decreased GABA release from parvalbumin-positive (PV) basket cells (Földy et al., 2013). A reduced probability of GABA release from PV-positive basket cells was detected also in layer IV somatosensory cortex (Cellot and Cherubini, 2014). Such deficit determines an alteration of the excitatory/inhibitory balance and a modification of the temporal window for integrating sensory signals. This may alter coherent percepts in autistic children.

Yuri Bozzi (Trento, Italy) reported a recent study on the neurofibromin-extracellular-regulated kinase (ERK) cascade in the hippocampus of WT and engrailed-2 knock-out (En2^−/−^) mice before and after spatial learning. The homeobox-containing transcription factor has been associated to ASD. En2^−/−^ mice show anatomical and behavioral “ASD-like” features, including loss of forebrain interneurons (Sgadò et al., 2013a), reduced expression of ASD-related genes (Sgadò et al., 2013b), decreased sociability, and learning deficits (Brielmaier et al., 2012). Deficits in signaling pathways involving neurofibromin and ERK have been associated to impaired learning. When compared to WT littermates, En2^−/−^ mice show impaired performance in the Morris water maze (MWM), associated with a marked down-regulation of neurofibromin expression in the hippocampus. ERK phosphorylation, known to be induced in the presence of neurofibromin deficiency, is increased in the hippocampus of En2^−/−^ mice after spatial learning. Treatment of En2^−/−^ mice with lovastatin, an indirect inhibitor of ERK phosphorylation, markedly reduced ERK phosphorylation in the dentate gyrus, but was unable to rescue learning deficits in MWM-trained mutant mice (Provenzano et al., 2014). Further investigation is needed to unravel the complex molecular mechanisms linking dysregulation of neurofibromin-dependent pathways to spatial learning deficits in the En2 mouse model of ASDs.

Yehezkel Ben-Ari (Marseille, France) discussed how understanding and treating neurological and psychiatric disorders is conditioned by a better knowledge of developmental processes. In the developing brain ionic currents, network activity, and molecular processes have unique features and follow a developmental sequence adapting them to their adult functions. Together with Spitzer (Ben-Ari and Spitzer, 2010), he proposed the checkpoint concept, according to which genes and neuronal activity cooperate in series to control the adequacy of the program implemented. In a previous study, the “neuroarcheology” theory was suggested (Ben-Ari, 2008). This consisted in the hypothesis that genetic or environmental insults may alter the developmental sequences producing pre-symptomatic architectural or electric signatures of disorders to come. In this perspective, neurons failing to implement their sequence, misplaced, or misconnected remain with immature features that are the factor impacting the operation of adjacent networks leading to the expression of neurological disorders. These concepts have been confirmed in relation to many early disorders (including infantile seizures, DCX mutations, TSC, nodular heterotopia, etc.). This also implies that future treatments will heavily rely on the use of selective drugs that antagonize specifically immature currents in the adult brain. These issues have been illustrated with recent clinical trials in autism successfully using the NKCC1 antagonist bumetanide (Lemonnier et al., 2012). The validity of this approach has been confirmed in two animal models of autism and fragile X where the diuretic ameliorated the behavioral and electrical features of autism (Tyzio et al., 2014).

Lindsay M. Oberman (Providence, RI, USA) highlighted recent studies showing the involvement of synaptic plasticity mechanisms in the pathophysiology of ASDs (Oberman et al., 2013). While most synaptic plasticity data in ASDs are derived from animal models, direct measures of circuit level plasticity in humans can be obtained by transcranial magnetic stimulation (TMS). This consists in stimulating focally the cortex with small intracranial electrical currents generated by a powerful and fluctuating extracranial magnetic field. TMS provide the only non-invasive method able to measure phenomena that closely resemble LTP and LTD in humans. Recently, patterned bursting protocols (i.e., theta burst stimulation, TBS), mimicking paradigms used to assess synaptic plasticity in acute rodent slices, have been developed. When applied to the motor cortex, continuous (cTBS), or intermittent TBS (iTBS) results in depression and potentiation of cortical reactivity as indexed through suppression and facilitation of motor evoked potentials (MEPs), respectively (Huang et al., 2010). Furthermore, published data suggest that cTBS leads to lasting suppression through enhancement of GABAergic inhibition (Benali et al., 2011). Both children and adults with ASDs exhibit abnormal plasticity mechanism and GABAergic dysfunctions (Oberman et al., 2010, 2012, 2013). Additional studies are underway to clarify the relationship between these indices and physiological and behavioral phenotype.

## Conflict of Interest Statement

The authors declare that the research was conducted in the absence of any commercial or financial relationships that could be construed as a potential conflict of interest.
